# Stress Echocardiography Beyond Wall Motion in ANOCA/INOCA: Coronary Microvascular Dysfunction and Multiparametric Assessment

**DOI:** 10.3390/jcdd13100500

**Published:** 2026-10-03

**Authors:** Nicole Carabetta, Pierfrancesco Durante, Assunta Di Costanzo, Martina Sportelli, Giovanni Canino, Antonio Strangio, Isabella Leo, Nadia Salerno, Jolanda Sabatino, Youssef S. Abdelwahed, Quirino Ciampi, Sabato Sorrentino, Salvatore De Rosa, Daniele Torella, Giuseppe Panuccio

**Affiliations:** 1 Department of Medical and Surgical Sciences, Magna Graecia University, 88100 Catanzaro, Italy; 2 Department of Cardiology, Angiology and Intensive Care Medicine, Deutsches Herzzentrum der Charité Berlin, 12200 Berlin, Germany; 3 Division of Cardiology, Fatebenefratelli Hospital Benevento, 82100 Benevento, Italy

**Keywords:** stress echocardiography, ANOCA, INOCA, coronary microvascular dysfunction, coronary flow velocity reserve, ABCDE protocol, vasospastic angina

## Abstract

Stress echocardiography has traditionally been used to identify inducible regional wall motion abnormalities caused by flow-limiting epicardial coronary artery disease. However, a substantial proportion of patients with angina or objective evidence of myocardial ischaemia have no obstructive coronary stenoses, highlighting the clinical relevance of angina and ischaemia with non-obstructive coronary arteries and, in particular, coronary microvascular dysfunction. In these patients, ischaemia may be diffuse, heterogeneous, and predominantly subendocardial with inducible regional wall motion abnormalities often absent or appearing only late in the ischemic cascade. This narrative review examines the evolving role of stress echocardiography beyond conventional wall-motion analysis, with particular emphasis on Doppler-derived coronary flow velocity reserve, multiparametric stress echocardiography and provocative approaches for vasomotor disorders. Coronary flow velocity reserve provides a non-invasive assessment of coronary vasodilator capacity and adds prognostic information beyond conventional stress imaging. Its integration with conventional wall-motion analysis and other stress echocardiographic parameters may provide a more comprehensive assessment of coronary vascular function, particularly in patients with ischemic symptoms and no obstructive coronary artery disease.

## 1. Introduction

Coronary artery disease (CAD) is a major cause of morbidity and mortality [1]. Atherosclerosis of epicardial coronary arteries is often responsible for CAD and its clinical symptoms, and coronary revascularization represents the cornerstone treatment for this clinical scenario [2]. Angina with non-obstructive coronary arteries (ANOCA) includes patients with symptoms consistent with myocardial ischaemia in whom no significant epicardial coronary stenoses are observed [3].

The related term ischaemia with non-obstructive coronary arteries (INOCA) should be reserved for patients in whom objective evidence of myocardial ischaemia has been demonstrated.

These conditions affect a substantial proportion of patients, and contemporary guidelines report that as many as 50–70% of patients with angina or suspected ischaemia referred to coronary angiography have no obstructive epicardial stenoses [4,5,6]. Importantly, ANOCA and INOCA should not be regarded as benign conditions. Beyond their high prevalence, these syndromes are associated with persistent symptoms, recurrent presentations to the emergency department and an increased risk of adverse cardiovascular outcomes [5,6]. Although contemporary consensus documents have substantially advanced the diagnostic framework of ANOCA/INOCA and broadened the role of stress echocardiography, the echocardiographic assessment of coronary vascular dysfunction remains fragmented across different techniques and physiological domains [7].

A focused literature search was conducted in PubMed/MEDLINE and Scopus from database inception to July 2026, using terms related to stress echocardiography, coronary microvascular dysfunction, ANOCA/INOCA, coronary flow velocity reserve and vasospastic angina. Original studies, relevant reviews, consensus documents, and guidelines were selected according to their clinical and scientific relevance to the scope of this review.

This work therefore provides an updated overview of stress echocardiography beyond conventional wall-motion analysis, with particular emphasis on coronary flow velocity reserve (CFVR), multiparametric assessment, vasomotor disorders and the dissociation between ischaemia and regional wall-motion abnormalities.

## 2. Pathophysiology of CMD

The underlying cause of symptoms in a significant percentage of INOCA patients is coronary microvascular dysfunction (CMD). Coronary microcirculation represents an essential component of the coronary circulation, accounting for a substantial proportion of coronary blood volume and playing a key role in the regulation of coronary blood flow [8].

Structural alterations include arteriolar remodelling, medial hypertrophy, perivascular fibrosis and capillary rarefaction, whereas functional abnormalities involve impaired vasodilatory capacity, endothelial dysfunction, increased microvascular resistance and abnormal vasomotor regulation. These mechanisms may coexist and contribute to myocardial ischaemia even in the absence of significant epicardial coronary stenosis [8,9].

Coronary microvascular dysfunction has also been described in heart failure with preserved ejection fraction, suggesting that microvascular impairment may extend beyond ischaemic syndromes [10].

The pathophysiology of CMD is complex; in particular, the main mechanism involves an impaired microvascular vasodilation, which results in an imbalance between myocardial oxygen supply and demand, expressed as reduced coronary flow reserve (CFR) [11].

Gold-standard techniques for measuring CFR are either invasive or involve ionizing radiation, limiting their widespread clinical adoption. For more than four decades, stress echocardiography was identified mainly with the detection of new or worsening regional wall motion abnormalities. Nevertheless, a stenosis centred interpretation captures only one component of the cardiovascular response to stress.

**Figure 1:**
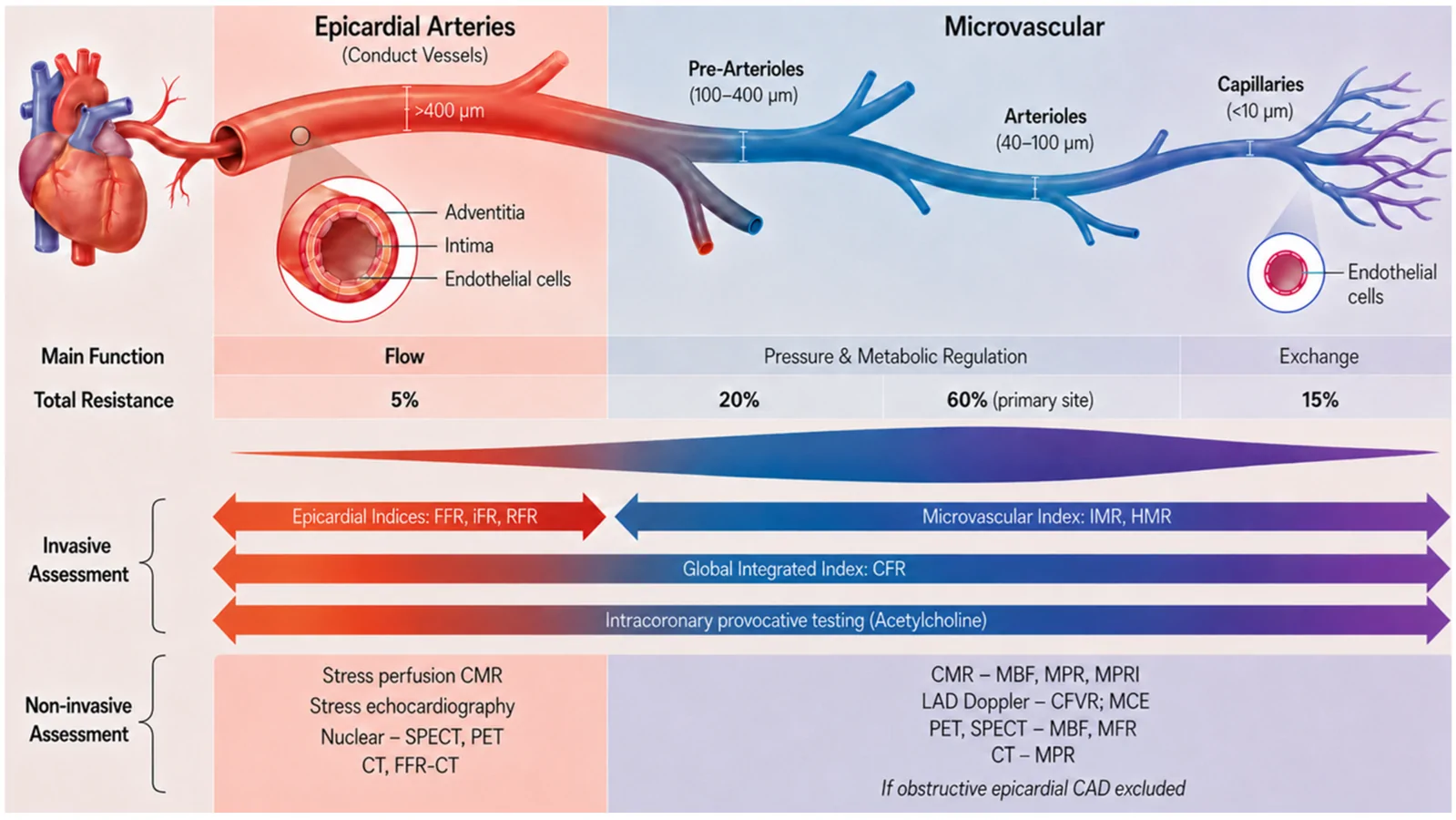
Physiological Features and Functional Assessment of Epicardial and Microvascular Coronary Compartments. Abbreviations: CAD, coronary artery disease; CFR, coronary flow reserve; CFVR, coronary flow velocity reserve; CMR, cardiac magnetic resonance; CT, computed tomography; FFR, fractional flow reserve; HMR, hyperaemic microvascular resistance; iFR, instantaneous wave-free ratio; IMR, index of microvascular resistance; LAD, left anterior descending; MBF, myocardial blood flow; MCE, myocardial contrast echocardiography; MFR, myocardial flow reserve; MPR, myocardial perfusion reserve; MPRI, myocardial perfusion reserve index; PET, positron emission tomography; RFR, resting full-cycle ratio; SPECT, single photon emission computed tomography.

The main physiological features of the epicardial and microvascular coronary compartments and the corresponding methods for their functional assessment are summarized in Figure 1.

In this context, impaired coronary vasodilator function can be assessed by transthoracic Doppler-derived CFVR, which has demonstrated both diagnostic and prognostic relevance [12].

Within this framework, the combined use of transthoracic Doppler-derived CFVR of the left anterior descending artery (LAD) and wall motion analysis enables a more complete evaluation of coronary physiology, providing a clinically relevant, non-invasive assessment of microvascular dysfunction. Overall, these developments support a shift toward a mechanism-based understanding of coronary vascular disease, enabling more precise phenotyping and potentially informing individualized clinical management [13].

## 3. Invasive Detection of Ischaemia

Invasive coronary physiology has therefore become the reference framework for the comprehensive functional characterization of coronary circulation, providing the physiological basis for understanding CMD and establishing the conceptual background for contemporary non-invasive strategies aimed at evaluating coronary vascular function [14,15].

The introduction of fractional flow reserve (FFR) represented a fundamental milestone in the evaluation of CAD, shifting clinical decision-making beyond anatomical stenosis assessment toward the characterization of its physiological significance. Unlike invasive coronary angiography, which provides morphological information regarding epicardial coronary lesions, FFR quantifies their functional impact by measuring the ratio between distal coronary pressure and aortic pressure during conditions of maximal pharmacological hyperaemia. Under conditions of minimized and relatively stable microvascular resistance during maximal hyperaemia, FFR estimates the proportion of maximal achievable myocardial blood flow preserved despite the presence of an epicardial stenosis [16].

An FFR value ≤ 0.80 identifies a haemodynamically significant epicardial coronary stenosis and has been adopted as the established threshold for guiding revascularization decisions in patients with stable coronary artery disease. Conversely, values > 0.80 generally indicate the absence of functionally significant flow limitation, although interpretation should consider the clinical context and the potential influence of diffuse coronary disease and microvascular abnormalities.

Values between 0.75 and 0.80 represent a diagnostic grey zone in which clinical presentation, ischemic burden and additional physiological information should guide decision-making.

Furthermore, FFR represents an integrated physiological measurement influenced by both epicardial stenosis severity and downstream microvascular conditions, particularly in the presence of abnormal coronary microvascular function. Therefore, abnormalities affecting the coronary microvasculature may influence FFR values and limit its ability to identify ischaemia caused by isolated microvascular abnormalities [17].

Consequently, although a normal FFR reliably excludes a flow-limiting epicardial stenosis, it does not exclude the presence of coronary microvascular dysfunction.

To overcome some of these limitations, non-hyperaemic pressure indices were subsequently developed, among which the instantaneous wave-free ratio (iFR) has become the most widely adopted. iFR evaluates the translesional pressure gradient during the wave-free period of diastole, a phase characterized by naturally low and relatively stable microvascular resistance, allowing physiological assessment without pharmacological vasodilation [18,19]. An iFR value ≤ 0.89 is considered indicative of a physiologically significant epicardial stenosis and represents the validated threshold used in contemporary revascularization strategies. These indices identify whether a stenosis is responsible for flow limitation but provide limited information regarding the functional integrity of the coronary microcirculation. This distinction is clinically relevant because many patients with angina and objective evidence of myocardial ischaemia exhibit normal epicardial physiology despite persistent symptoms. In these patients, ischemic mechanisms frequently originate from abnormalities beyond epicardial coronary arteries, highlighting the need for complementary physiological parameters specifically designed to evaluate microvascular function [20].

Among available invasive physiological parameters, coronary flow reserve (CFR) represents the most established measure of the functional capacity of the coronary circulation. CFR is defined as the ratio between maximal hyperaemic coronary blood flow and resting coronary blood flow and reflects the ability of the coronary vascular system to increase perfusion in response to increased myocardial metabolic demand [21].

A preserved CFR indicates adequate vasodilatory capacity, whereas a reduced CFR identifies an impaired ability of the coronary circulation to appropriately augment blood flow during conditions of increased demand. In clinical practice, a CFR value below 2.0 is generally considered indicative of impaired coronary vasodilatory capacity, although the optimal threshold may vary according to the methodology used, patient characteristics and clinical setting. Importantly, reduced CFR has demonstrated prognostic relevance across different cardiovascular conditions, including patients with and without obstructive coronary artery disease, highlighting that coronary vascular dysfunction represents an independent determinant of cardiovascular risk beyond the presence of epicardial atherosclerosis [22].

However, CFR should not be interpreted as a specific marker of microvascular dysfunction alone. Since it represents an integrated assessment of the entire coronary circulation, CFR is influenced by both epicardial and microvascular compartments. Therefore, a reduced CFR may result from flow-limiting epicardial stenosis, diffuse atherosclerotic disease, endothelial dysfunction, increased microvascular resistance or a combination of these mechanisms [22].

A reduced CFR identifies an abnormal coronary circulatory reserve but requires integration with additional physiological parameters to determine whether the predominant mechanism is increased microvascular resistance, endothelial dysfunction, diffuse epicardial disease or a combination of these abnormalities.

To overcome this limitation, the index of microcirculatory resistance (IMR) was introduced as a more specific invasive parameter of coronary microvascular function. IMR quantifies minimal microvascular resistance during pharmacologically induced hyperaemia by combining distal coronary pressure measurements with coronary transit time, providing a relatively direct assessment of the microvascular compartment [23].

Unlike CFR, IMR is less influenced by resting haemodynamic conditions and provides a more specific evaluation of microvascular resistance, particularly in the absence of significant epicardial coronary disease. An IMR value ≥ 25 is generally considered indicative of abnormal microvascular resistance and has been associated with adverse clinical outcomes. Values ≥ 40 have been associated with more severe microvascular impairment and poorer prognosis, particularly after acute myocardial infarction [11].

However, interpretation of IMR requires awareness of potential technical limitations, including dependence on accurate coronary transit time measurement, influence of collateral circulation and possible confounding effects of diffuse epicardial disease. Increased IMR has been identified in patients with ANOCA and INOCA, supporting its role in the identification of patients with impaired microvascular function despite normal epicardial coronary arteries [24].

Beyond IMR, other invasive indices, such as hyperaemic microvascular resistance (HMR), based on Doppler-derived coronary flow velocity, have been proposed but remain mainly confined to specialized invasive laboratories [25].

In addition to impaired vasodilatory capacity and increased microvascular resistance, coronary dysfunction may result from abnormalities in vasomotor regulation. In this context, acetylcholine provocation testing allows the evaluation of endothelial-dependent coronary responses by assessing the ability of coronary vessels to appropriately regulate vascular tone. In healthy coronary arteries, acetylcholine induces endothelium-dependent vasodilation through nitric oxide release. However, in the presence of endothelial dysfunction, this response may be impaired or reversed, resulting in paradoxical vasoconstriction. Acetylcholine testing can therefore identify abnormal coronary vasomotor responses that remain undetected by conventional angiography, FFR, CFR and IMR measurements [26,27].

The WISE program further demonstrated the clinical relevance of coronary microvascular dysfunction, showing that impaired coronary microvascular reactivity to adenosine is common in women with suspected ischaemia and no obstructive artery disease and is associated with an increased risk of adverse cardiovascular outcomes [28].

The integration of CFR, IMR, HMR and acetylcholine testing allows identification of distinct CMD phenotypes, including impaired vasodilatory reserve, increased microvascular resistance and abnormal vasomotor responses, which may coexist within the same patient but require different therapeutic strategies.

The clinical relevance of this phenotype-based approach was further supported by the CorMicA trial, in which invasive identification of microvascular and vasospastic endotypes followed by stratified medical therapy resulted in significant improvements in angina and quality of life compared with standard care [29].

The main invasive indices used to assess epicardial and microvascular coronary function are summarized in Table 1.

## 4. Stress Echocardiography

Despite its diagnostic accuracy and mechanistic relevance, invasive coronary function testing remains limited by its invasive nature, the requirement for specialized expertise and the need for pharmacological provocation protocols. These limitations have stimulated the development of non-invasive strategies aimed at identifying functional abnormalities of the coronary circulation and translating the principles derived from invasive physiology into clinical practice.

In this context, the relationship between invasive physiology and functional imaging should be considered complementary rather than competitive. Invasive assessment defines the underlying mechanism responsible for coronary dysfunction, whereas non-invasive techniques aim to detect its functional expression and clinical consequences. This integrated approach represents a shift from a disease model centred exclusively on anatomical stenosis toward a comprehensive evaluation of coronary vascular function.

The principal non-invasive modalities available for the assessment of coronary microvascular function are summarized in Table 2.

In cardiology, stress echocardiography is a widely accessible and non-invasive diagnostic technique. Despite these benefits, it is still not widely used to diagnose CMD.

Doppler echocardiography and myocardial contrast echocardiography (MCE) are two stress TTE methods that have demonstrated potential in the diagnosis of CMD.

MCE employs lipid microbubbles (less than 10 μm) with high echogenicity. The small size makes it possible to move through the microvasculature, by improving the visualization of contrast agent perfusion through the myocardium. Contrast is infused continuously and the microbubbles are destroyed with the use of high-frequency ultrasound. The replenishment of microbubbles is then used to quantify a time-intensity curve, from which the volume of myocardial blood flow is calculated.

Studies have demonstrated that patients with cardiac syndrome X have significantly lower MBF during MCE at rest and after vasodilation with adenosine. This finding demonstrates that MCE can identify CMD in individuals with nonobstructive CAD [30].

Another echocardiographic method that can be utilized to diagnose CMD is Doppler echocardiography with CFVR. Doppler-derived CFVR has been recognized as a suitable non-invasive approach in selected patients with suspected INOCA, in fact it has shown good agreement with PET [13].

Transthoracic Doppler echocardiography measures diastolic coronary flow velocity in the mid-distal left anterior descending artery at rest and during pharmacological vasodilation. CFVR is calculated as the ratio of peak hyperaemic to resting diastolic velocity. In patients in whom obstructive epicardial coronary disease has been excluded, a reduced CFVR is interpreted as evidence of impaired coronary microcirculation and is commonly used as a non-invasive marker of CMD, as it reflects the ability of the coronary circulation to increase blood flow in response to vasodilator stress.

A threshold of 2.0 is frequently adopted, although values between 2.0 and 2.5 should be interpreted as a physiological grey zone influenced by age, haemodynamics, medications, caffeine exposure, left ventricular hypertrophy and stress protocol [31].

A reduced CFVR has been reported in approximately 25–30% of patients with angina and non-obstructive coronary arteries, with prevalence varying according to patient selection and methodology [32,33].

In this setting, regional wall motion abnormalities (RWMAs) are often absent, since microvascular ischaemia typically has a diffuse rather than a segmental distribution [33].

The clinical importance of reduced CFVR extends beyond diagnosis. Doppler-derived CFVR has shown incremental prognostic value over conventional stress imaging and myocardial perfusion assessment, and predicts cardiovascular events in patients with angina and no obstructive coronary artery disease [34,35].

## 5. Multiparametric Stress Echocardiography: From Ischaemia Detection to Functional Phenotyping

The traditional stress echocardiographic examination has been centred on the detection of inducible RWMA, a highly specific marker of flow-limiting epicardial coronary artery disease. This approach remains clinically valuable, but it interrogates only the mechanical end point of the ischaemic cascade and therefore incompletely characterizes patients whose symptoms arise from diffuse atherosclerosis, CMD, abnormal loading conditions, impaired contractile reserve, or autonomic dysfunction. Contemporary stress echocardiography has consequently evolved from a single marker test into a multiparametric physiological examination [7,36].

The ABCDE protocol embodies this transition by assessing five complementary domains during the stress study: regional wall motion (step A), pulmonary congestion (step B), left ventricular contractile reserve (step C), coronary flow velocity (D) and heart-rate reserve (E) [37].

Step A remains the indispensable foundation of stress echocardiography and primarily reflects the ability of epicardial coronary flow to meet myocardial demand. A new or worsening abnormality involving at least two contiguous segments is considered the hallmark of inducible ischaemia, whereas a fixed abnormality suggests scar and a low-dose improvement followed by deterioration at higher stress indicates a biphasic response [7,36].

Step B extends the examination beyond the myocardium by using ultrasound to identify stress-induced extravascular lung water. B-lines are vertical artefacts arising from the pleural line, extending to the far field without fading, and moving synchronously with respiration. The physiological meaning of step B is an inadequate diastolic and haemodynamic adaptation to stress. Impaired relaxation, reduced chamber compliance, dynamic mitral regurgitation or limited preload reserve may increase pulmonary capillary pressure and generate interstitial oedema. B-line assessment is highly feasible during stress echocardiography and can be performed rapidly at both rest and peak stress. Importantly, stress-induced B-lines are more frequent than resting B-lines, suggesting that exercise may unmask latent pulmonary congestion that is not evident at baseline and may occur across different cardiovascular conditions, including heart failure with preserved or reduced ejection fraction and valvular heart disease [38,39].

Step C evaluates the capacity of the left ventricle to increase systolic performance. The normal response is an increase in ejection fraction accompanied by a reduction in end-systolic volume. Left ventricular force, commonly approximated as systolic blood pressure divided by ESV, can be calculated at baseline and peak stress; the peak-to-rest force ratio has been used as an index of contractile reserve. In the setting of ANOCA or CMD, this step is particularly useful for distinguishing preserved regional wall motion with normal contractile reserve from a more diffuse myocardial disease with a reduced contractile reserve [7,40].

Among the ABCDE components, step D is the most directly related to coronary vascular physiology. CFVR integrates the ability of both epicardial and microvascular compartments to increase flow. When obstructive epicardial coronary artery disease has been excluded, however an impaired CFVR provides non-invasive evidence of reduced coronary vasodilator capacity.

In a large multicentre stress echocardiography cohort, CFVR is feasible in 88% of patients, and a value of 2.0 or lower independently predicted adverse events beyond RWMA. In women with angina and no obstructive coronary disease, the iPower study further demonstrated that Doppler-derived CFVR was independently associated with cardiovascular outcomes [33,41].

The final component is imaging-independent: heart-rate reserve (HRR), calculated as the ratio of peak to resting heart rate. Chronotropic incompetence (CI) is broadly defined as the inability of the heart to adequately increase its rate in response to metabolic demands during exercise. Its assessment during comprehensive stress echocardiography provides insight into cardiac autonomic dysfunction. A blunted HRR reflects impaired sympathetic activation or broader cardiac autonomic dysfunction and has been associated with adverse prognosis independently of inducible RWMA. During dipyridamole stress, an HRR of 1.22 or lower predicted cardiac events, including myocardial infarction even in patients receiving beta blockers, and its combination with CFVR identified a high-risk group of patients. In patients with chronic coronary syndromes, chronotropic incompetence during exercise or pharmacological stress is a simple and objective predictor of survival [42,43,44].

In a prospective multicentre validation study of 3754 patients, increasing numbers of abnormal steps were associated with progressively worse survival, and the multiparametric model improved risk prediction beyond clinical variables and wall motion analysis. Moreover, the overall ABCDE score may help differentiate obstructive coronary artery disease from ANOCA. In a multicentre cohort, ANOCA was more likely in patients with an ABCDE score ≤ 2, whereas the probability of significant CAD progressively increased with higher scores; with scores of 4 and 5, CAD was approximately 2-fold and 14-fold more likely than ANOCA, respectively [45].

In this sense, multiparametric stress echocardiography represents the transition from ischaemia detection to functional imaging [37].

## 6. The Dissociation Between Ischaemia and Wall Motion in CMD

In coronary microvascular dysfunction, stress testing often causes angina and ST-segment depression, in the absence of regional wall motion abnormalities. This pattern differs from the conventional ischemic cascade associated with flow-limiting epicardial stenosis [30].

A plausible explanation could be the development of small, patchy areas of predominantly subendocardial ischaemia, resulting from increased resistance at the pre-arteriolar or intramural level, which is not detectable by coronary angiography. Accordingly, preserved left ventricular wall motion during stress echocardiography does not exclude ischaemia; regional dysfunction generally becomes visible when the ischemic burden exceeds a critical threshold, approximately 20% of transmural wall thickness and 5% of total myocardial mass; more limited and scattered subendocardial ischaemia may therefore produce symptoms, diastolic dysfunction and electrocardiographic changes without inducing detectable RWMA.

In CMD, blunted augmentation of global longitudinal strain may occur even when radial thickening remains preserved. This reflects the greater vulnerability of longitudinal, predominantly subendocardial fibers. Accordingly, in INOCA, impaired resting GLS or a reduced strain reserve during stress may support the presence of stress-induced ischaemia [46,47,48].

## 7. Vasospastic Angina

Vasospastic angina is caused by transient, dynamic vasoconstriction involving the epicardial coronary arteries, microcirculation or both.

Epicardial coronary vasospasm represents another potential mechanism of ischaemia in INOCA. It may be incidentally provoked by exercise, dobutamine, or dipyridamole and can produce transmural ischaemia with regional wall motion abnormalities [49].

Alternatively, vasospasm may be specifically investigated using provocative protocols, such as ergonovine or hyperventilation stress echocardiography.

Several studies have shown that non-invasive provocative stress testing may help identify epicardial coronary spasm through the development of ischemic electrocardiographic changes and/or transient regional wall motion abnormalities. In addition, transthoracic Doppler assessment of coronary flow during different stress protocols may provide complementary information on functional coronary abnormalities in patients with angina and non-obstructive coronary arteries, including impaired microvascular dilatation and abnormal vasoconstrictor responses. More recently, in patients with ANOCA, transthoracic Doppler assessment of coronary flow during functional testing has been reported to correlate with invasive coronary function testing, further supporting its role as a complementary non-invasive approach for the assessment of coronary microvascular function [50,51,52].

Ergonovine is the most established non-invasive pharmacological provocation agent and induces coronary constriction through serotonergic and alpha-adrenergic pathways. An ischemic response is characterized by reversible regional wall abnormalities that could precede ST-segment changes. Large observational data suggest that ergonovine stress echocardiography can be performed with a low complication rate in experienced centres [53,54].

Furthermore, another stressor that could be used in this context is hyperventilation.

Hypocapnic alkalosis increases vascular smooth-muscle reactivity, with positivity most commonly emerging during the first minutes of recovery. In the transthoracic echocardiography subgroup of a recent meta-analysis, hyperventilation testing showed a pooled sensitivity and specificity of approximately 90% and 98%, respectively.

Its sensitivity may be enhanced by combining hyperventilation with exercise [50,55,56].

Simultaneous assessment of wall motion and coronary flow may also explain the mechanism of vascular spasm, in particular, wall motion abnormalities associated with severe reduction or absence of coronary flow are suggestive of epicardial coronary spasm, while a blunted or paradoxically reduced flow response associated with angina but without wall motion abnormalities may raise the suspicion of a predominantly microvascular vasomotor mechanism; however, this pattern should not be regarded as a substitute for invasive coronary function testing [57].

## 8. Clinical and Practical Implications of Stress Echocardiography in CMD

In patients with persistent angina or suspected myocardial ischaemia and no obstructive coronary artery disease, conventional wall-motion analysis may underestimate the presence of ischaemia, as CMD frequently occurs without inducible regional wall motion abnormalities [30,31,33].

In clinical practice, the overall ABCDE profile may further refine patient phenotyping. Lower ABCDE scores are more commonly associated with ANOCA, whereas the likelihood of significant epicardial coronary artery disease progressively increases with higher scores. In addition, the number of abnormal ABCDE components provides prognostic information, with progressively worse outcomes as the burden of abnormal parameters increases [37,45].

Stress echocardiography may also provide useful clinical clues when a vasospastic mechanism is suspected. Epicardial coronary spasm may result in transient regional wall motion abnormalities during provocative stress, whereas angina and ischemic changes associated with an abnormal coronary flow response but preserved wall motion may raise the suspicion of a predominantly microvascular vasomotor disorder. In selected patients, provocative approaches may therefore complement conventional stress echocardiography. However, these findings should be interpreted within the overall clinical context, and invasive coronary function testing remains the reference method for definitive characterization of coronary vasomotor endotypes [54,56,57].

Beyond diagnosis, stress echocardiography may also contribute to risk stratification. Moreover, with the multiparametric ABCDE approach, prognosis progressively worsens with the increasing number of abnormal components. Overall, these findings highlight the clinical and prognostic value of stress echocardiography in the assessment of patients with suspected CMD [33,37,41].

## 9. Conclusions

The future of stress echocardiography will depend less on the addition of isolated indices than on the integration of quantitative parameters into a coherent physiological profile. Contemporary multiparametric protocols already extend interpretation beyond inducible regional wall motion abnormalities. In patients with ANOCA and INOCA, this approach may help characterize overlapping mechanisms such as coronary microvascular dysfunction, vasomotor abnormalities, and impaired myocardial or autonomic reserve. Artificial intelligence is likely to facilitate this transition through automated segmentation, quantification of ejection fraction and global longitudinal strain, and recognition of wall-motion abnormalities, with early studies showing improved diagnostic performance and reduced inter-operator variability [58,59].

Stress-derived deformation and myocardial work may identify subtle systolic abnormalities before conventional wall motion changes become evident.

In conclusion, stress echocardiography should no longer be considered only a test to detect obstructive epicardial coronary artery disease. By integrating wall motion with coronary flow, myocardial reserve, pulmonary congestion, autonomic response and selected provocative protocols such as ergonovine or hyperventilation, stress echocardiography can provide a comprehensive and accessible evaluation together with individualized risk assessment.

## Figures and Tables

**Table 1:** Invasive assessment of epicardial and microvascular compartments.

Index	Definition	Measures	Threshold	Clinical Interpretation in CMD	Limitations
FFR	The ratio of distal coronary pressure to aortic pressure (Pd/Pa) during hyperemia	Epicardial stenosis severity	≤0.80	Identifies haemodynamically significant epicardial stenosis	- Does not directly assess microvascular function - A normal FFR does not exclude CMD - May not be reliable in conditions with severe microvascular dysfunction (e.g., acute MI)
iFR	The ratio of distal coronary pressure to aortic pressure (Pd/Pa) during the wave-free diastolic period	Epicardial stenosis severity without pharmacological hyperaemia	≤0.89	Identifies haemodynamically significant epicardial stenosis	May not be reliable in conditions with severe microvascular dysfunction (e.g., acute MI)
CFR	Doppler-derived or thermodilution-derived coronary flow assessment during hyperaemia	Epicardial stenosis severity and microvascular resistance	CFR < 2.0; values between 2.0 and 2.5 may represent a method-dependent grey zone	Reduced CFR indicates impaired global coronary vasodilatory reserve When severe obstructive disease of the epicardial arteries is ruled out, reduced CFR is one of the hallmarks of CMD	- Not specific to the microcirculation - Does not identify the underlying mechanism of CMD - Technique- and protocol-dependent - Affected by hemodynamic conditions - Thermodilution methods may overestimate CFR
IMR	Product of the distal coronary pressure and mean transit time of a saline bolus during maximal hyperaemia	Microvascular resistance	≥25	More specific for microvascular resistance and less influenced by resting haemodynamics than CFR	- Administration of intravenous adenosine - Indirect measure of microvascular resistance
HMR	Pressure distal to a stenosis divided by the distal average peak velocity during maximal hyperaemia, during the whole cardiac cycle	Microvascular resistance	≥1.9–2.5 (mmHg·cm·s)	Increased HMR supports abnormal microvascular resistance	- Less standardized than IMR - Technically demanding and operator-dependent - Limited availability - Variable cut-offs across studies
Intracoronary provocative testing (Acetylcholine)	Graded intracoronary acetylcholine with simultaneous angiography, ECG and symptom assessment	Endothelium-dependent coronary vasomotor function and susceptibility to epicardial and microvascular vasospasm	Microvascular spasm: symptoms and ischemic ECG changes, but no epicardial spasm during acetylcholine testing	Differentiates epicardial vasospastic angina from microvascular vasospasm	- Additional contrast and radiation - Risk of arrhythmias - Lack of availability

Abbreviations: CFR, coronary flow reserve; CMD, coronary microvascular dysfunction; FFR, Fractional flow reserve; HMR, Hyperaemic Microvascular Resistance; iFR, instantaneous wave-free ratio; IMR, index of microvascular resistance; MI, myocardial infarction.

**Table 2:** Non-invasive assessment of coronary microvascular dysfunction.

Modality	Technique	Agent	Parameter	Abnormal Threshold	Advantages	Limitations
Stress Echocardiography	Doppler flow velocity recordings at rest and during vasodilator stress in the LAD artery	Adenosine Dipyridamole Regadenoson	CFVR	CFVR ≤ 2.0	- Bedside procedure - Inexpensive - No radiation exposure - High feasibility - Good correlation with invasive measurement - Potential MBF quantification with MCE, despite limited standardization	- Limited to LAD region - Extensive training - Operator dependence - Technical pitfalls (poor acoustic windows, obesity, lung disease) - Obstructive CAD needs to be excluded - Influenced by age, haemodynamics, LV hypertrophy, medications, caffeine exposure and stress protocol
PET	Dynamic myocardial perfusion imaging at rest and during vasodilator stress	Stress agent (Adenosine, Dipyridamole, Regadenoson) Perfusion radiotracer (^15^O-water, ^13^N-ammonia, ^82^Rb, ^18^F-labelled agents)	MBF ^b^ MFR ^c^	Reduced hyperaemic MBF (<1.7–2.3 mL/min/g, method-dependent) MFR < 2.0	- Gold standard for non-invasive assessment of coronary microvascular function - Evaluates global and regional MBF - Good correlation with invasive measurement - Prognostic value - Good reproducibility - Not limited by renal function	- Limited availability - Limited spatial resolution - Expensive - Time consuming procedure - Radiation exposure - Dedicated tracers/software and standardized acquisition - Obstructive CAD needs to be excluded
CMR	First-pass myocardial perfusion imaging at rest and during vasodilator stress	Stress agent (Adenosine, Dipyridamole, Regadenoson) Gadolinium-based contrast agents	MPR MBF MPRI	MPRI < 2.0 Quantitative MBF/MPR thresholds are technique-dependent and not universally standardized	- High spatial resolution - Tissue characterization - No radiation exposure - Validated and compared with PET and invasive methods	- Limited availability - Expensive - Time consuming procedure - Imaging artifacts - Patient compliance - Possible contraindications to CMR [e.g., nonconditional pacemakers and ICDs] or to gadolinium-based contrast agents (e.g., renal failure due to the potential risk of nephrogenic systemic fibrosis)
CT scan	ECG-triggered first-pass myocardial perfusion imaging at rest and during vasodilator stress	Vasodilator stress agent (Adenosine Dipyridamole Regadenoson) Iodine-based contrast agent	MBF MPR	MPR < 2	- Exclusion of obstructive epicardial CAD - High spatial resolution - CTA-derived FFR	- Radiation exposure - Risk of contrast-associated kidney injury - Potential MBF overestimation due to contrast-induced vasodilation
SPECT	Dynamic myocardial perfusion imaging at rest and during vasodilator stress	Stress agent (Adenosine, Dipyridamole, Regadenoson) Radiotracers (^99m^Tc-Sestamibi, ^99m^Tc-Tetrofosmin)	MBF and MFR (with CZT-SPECT)	Non-standardized cut-off values	- Wide availability - Lower cost than PET - Quantification of MBF and MFR with dynamic CZT-SPECT - Shorter acquisition time and lower radiation dose with newer CZT systems - Simultaneous relative perfusion assessment	- Radiation exposure - Lower spatial resolution than PET/CMR - Conventional SPECT assesses relative rather than absolute perfusion - Limited availability of dynamic CZT systems - Tracer extraction limitations - Lack of standardized acquisition, reconstruction, software, and CMD-specific thresholds

Abbreviations: CAD, Coronary artery disease; CFVR, Coronary flow velocity reserve; CMD, Coronary microvascular dysfunction; CMR, cardiac magnetic resonance; CT, computed tomography; CZT, cadmium–zinc–telluride; FFR, fractional flow reserve; ICDs, implantable cardioverter defibrillators; LAD, Left anterior descending; MBF, myocardial blood flow; MCE, myocardial contrast echocardiography; MFR, myocardial flow reserve; MPR, myocardial perfusion reserve; MPRI, myocardial perfusion reserve index; PET, Positron emission tomography; SPECT, single photon emission computed tomography. b = expressed as millilitres per minute per gram of myocardium; c = stress MBF/rest MBF.

## Data Availability

No new data were created or analyzed in this study. Data sharing is not applicable to this article.

## Abbreviations

CMD: coronary microvascular dysfunction; CAD: coronary artery disease; FFR: fractional flow-reserve; iFR: instantaneous-wave free ratio; CFR: coronary flow reserve; CFVR: coronary flow velocity reserve; IMR: index of microcirculatory resistance; HMR: hyperaemic microvascular resistance; RWMA: regional wall motion abnormalities; GLS: global longitudinal strain.
